# Supplementary material for: Trust in Generative AI for Health Information Consumption and the Effect of Learned Dependency: Randomized Controlled Experimental Study
Source: J Med Internet Res. 2026 Sep 16;28:e98326. doi: 10.2196/98326 (PMC13580606; doi:10.2196/98326)
Supplement: Supplementary file 1 [file jmir-v28-e98326-s002.pdf]

CHERRIES Checklist

Checklist for Reporting Results of Internet E-Surveys (Eysenbach, 2004)

Manuscript: Trust in Generative AI for Health Information Consumption and the Effect of Learned Dependency: An Experimental Study

Authors: Arif Ahmed, Gondy Leroy, Agrim Sachdeva, Philip Harber, Stephen A. Rains, Seokjun Youn, Prosanta Barai

Note. This checklist covers the online survey/experiment administered via Qualtrics for both experiments. Where responses differ between experiments, both are reported separately.

Item Category	Checklist Item	Response / Location in Manuscript
**DESIGN**		
Design	*Describe survey design, target population, sample frame. Is it a convenience sample?*	Experiment 1: Convenience sample of 338 junior-level business students at the University of Arizona, recruited via course credit. Experiment 2: Closed survey of 563 US-based Amazon Mechanical Turk (MTurk) workers with ≥95% approval rating. Both experiments used a 2×2 between-subjects factorial design manipulating information accuracy (correct vs. incorrect) and visual attention cues (highlight vs. no highlight). See Methodology section.
**IRB APPROVAL AND INFORMED CONSENT**		
IRB Approval	*Was the study approved by an IRB?*	Approved by the University of Arizona IRB prior to data collection (Protocol: STUDY00002235, determined Exempt). See Ethical Considerations section. IRB consent documentation included as supplementary file.
Informed Consent	*Describe the informed consent process.*	All participants provided informed consent before beginning the survey. Consent covered: study purpose, estimated completion time (~10 minutes), voluntary participation, data storage on Qualtrics accessible only to the research team. Full consent form provided as supplementary file.
Data Protection	*What mechanisms protected unauthorized access to personal data?*	No personally identifiable information was collected. Data stored securely on the Qualtrics platform (https://uarizona.co1.qualtrics.com), accessible only to the research team. Participants identified only by Qualtrics-assigned response IDs.
**DEVELOPMENT AND PRE-TESTING**		
Development and Testing	*How was the survey developed? Was usability and technical functionality tested?*	Survey developed in Qualtrics with embedded randomization logic. Five MIMIC-III clinical discharge summaries were selected and paired with GPT-4o-generated counterparts. Accurate and inaccurate versions were prepared by the research

		team (3 altered locations in Experiment 1; 6 in Experiment 2). Bold-text highlighting applied programmatically in the visual cue condition. Survey was internally piloted by the research team to verify randomization, display, and back-navigation disabling before deployment.
**RECRUITMENT PROCESS AND SAMPLE ACCESS**		
Open vs. Closed Survey	*Was it an open (any visitor) or closed (known sample) survey?*	Experiment 1: Closed survey as link distributed only to enrolled students. Experiment 2: Closed survey as restricted to US-based MTurk workers with ≥95% approval rating via the MTurk platform.
Contact Mode	*Was initial contact made online or offline?*	Experiment 1: Offline initial contact (course instructor announcement); survey accessed online via Qualtrics. Experiment 2: Online contact through the Amazon Mechanical Turk platform.
Advertising the Survey	*How/where was the survey announced or advertised?*	Experiment 1: Announced by course instructor to enrolled junior-level business students; 10 extra credit points offered for completion. Experiment 2: Posted as a Human Intelligence Task (HIT) on Amazon Mechanical Turk with $0.50 compensation. Eligibility restricted to US-based workers with ≥95% approval rating.
**SURVEY ADMINISTRATION**		
Web/E-mail	*State the type of e-survey.*	Web-based survey hosted on Qualtrics. Responses captured automatically; no manual data entry.
Context	*Describe the website or context in which the survey was posted.*	Experiment 1: Survey link distributed via course management system. Experiment 2: Survey posted as a HIT on Amazon Mechanical Turk (www.mturk.com), a widely used crowdsourcing platform for behavioral research. MTurk workers access tasks voluntarily through worker accounts.
Mandatory/Voluntary	*Was participation mandatory or voluntary?*	Voluntary in both experiments. Experiment 1: Extra credit incentive offered but participation was not required for course completion. Experiment 2: Workers voluntarily chose to accept and complete the HIT.
Incentives	*Were any incentives offered?*	Experiment 1: 10 extra credit points. Experiment 2: $0.50 monetary compensation via MTurk.
Time/Date	*What was the timeframe for data collection?*	Both experiments conducted in 2024. Specific dates available from the corresponding author upon request.
Randomization of Items or Questionnaires	*Were items or questionnaires randomized?*	Yes. Qualtrics automatically randomized participants across 20 experimental groups (2 accuracy conditions × 2 visual cue conditions × 5

		health texts). Back-navigation was disabled to prevent order effects.
Adaptive Questioning	*Was adaptive questioning used?*	No adaptive questioning. All participants completed the same survey structure with condition determined by randomization.
Number of Items	*How many questionnaire items per page?*	Survey comprised: (1) Demographic questionnaire; (2) GenAI dependency scale (6 items in Experiment 1; 17 items in Experiment 2); (3) Experimental stimulus display (source text + AI-generated text side-by-side); (4) Trust scale (3 items in Experiment 1; 4 items in Experiment 2 after CFA refinement).
Number of Screens (Pages)	*Over how many pages was the questionnaire distributed?*	Approximately 4 main survey sections presented across multiple Qualtrics pages. Total estimated completion time: ~10 minutes (maximum time limit enforced: 20 minutes).
Completeness Check	*Were completeness or consistency checks performed before submission?*	Qualtrics forced response was enabled, requiring responses to all items before advancing. Participants with missing data on any study variable were excluded via listwise deletion prior to analysis, yielding final samples of N=338 (Experiment 1) and N=563 (Experiment 2).
Review Step	*Were respondents able to review and change answers?*	No. Back-navigation was disabled in Qualtrics to prevent participants from returning to previous pages and revising responses.
**RESPONSE RATES**		
Unique Site Visitor	*How were unique visitors determined?*	Experiment 1: Qualtrics session-based tracking; course-based recruitment ensured one response per student. Experiment 2: MTurk platform enforces one submission per worker per HIT.
View Rate	*Ratio of unique survey visitors / unique site visitors.*	Not applicable for Experiment 1 (course-based closed recruitment). For Experiment 2: not directly measurable as MTurk does not provide data on workers who viewed but did not accept the HIT.
Participation Rate	*Ratio of visitors who agreed to participate / first-page visitors.*	Not directly measurable. Experiment 1: all students informed who chose to participate were enrolled. Experiment 2: participation determined by MTurk workers accepting the HIT.
Completion Rate	*Ratio of users who finished the survey / users who agreed to participate.*	Experiment 1: High completion due to course credit incentive. Experiment 2: MTurk workers who accepted the HIT but did not submit were not included in the dataset. Final samples after listwise deletion: N=338 (Experiment 1), N=563 (Experiment 2).
**PREVENTING MULTIPLE ENTRIES**		

Cookies Used	*Were cookies used to assign unique user identifiers?*	Qualtrics assigns unique response IDs to each session and default cookie-based controls were enabled. Experiment 2: MTurk platform-level controls prevent the same worker from submitting a HIT twice.
IP Check	*Was the IP address used to identify potential duplicate entries?*	Not explicitly used beyond Qualtrics default session management. Experiment 2: MTurk platform controls prevent duplicate submissions at the account level.
Log File Analysis	*Were log files analyzed for duplicate entries?*	Not applicable. Duplicate prevention handled at the platform level (Qualtrics session management; MTurk HIT submission controls).
Registration	*For closed surveys, how was duplicate submission prevented?*	Experiment 1: Survey link distributed to enrolled students; Qualtrics session controls prevented re-entry. Experiment 2: MTurk worker accounts prevent duplicate HIT submissions at the platform level.
**ANALYSIS**		
Handling of Incomplete Questionnaires	*Were only completed questionnaires analyzed? How were incomplete submissions handled?*	Only fully completed questionnaires were analyzed. Participants with missing data on any study variable were excluded via listwise deletion. Forced response in Qualtrics minimized item-level missingness. Final analytic samples: N=338 (Experiment 1), N=563 (Experiment 2). See Data Analysis section and Data Availability Statement.
Questionnaires Submitted with Atypical Timestamp	*Were fast-submission questionnaires excluded?*	No formal response time exclusion criterion was applied. A maximum time limit of 20 minutes was enforced in Qualtrics. MTurk workers are experienced survey participants; implausibly fast responses were not identified as a concern in the final datasets.
Statistical Correction	*Were weighting or propensity scores used to adjust for non-representative sampling?*	No statistical correction for non-representative sampling was applied. Both samples are acknowledged as convenience samples. The student sample limitation (Experiment 1) is discussed in the Limitations section. Cross-sample replication across two distinct samples provides convergent evidence. See Limitations and Future Directions sections.

Reference: Eysenbach G. Improving the Quality of Web Surveys: The Checklist for Reporting Results of Internet E-Surveys (CHERRIES). J Med Internet Res. 2004;6(3):e34. doi:10.2196/jmir.6.3.e34
