# Supplementary material for: Trust in Generative AI for Health Information Consumption and the Effect of Learned Dependency: Randomized Controlled Experimental Study
Source: J Med Internet Res. 2026 Sep 16;28:e98326. doi: 10.2196/98326 (PMC13580606; doi:10.2196/98326)
Supplement: Supplementary file 2 [file jmir-v28-e98326-s001.docx]

**APPENDIX A: Summary of Measures**

Our study used several validated and adapted measures embedded within a structured Qualtrics instrument. Below, we summarize the items used for the trust in GenAI and GenAI dependency measures for both experiments.

**Trust in GenAI Items**

Experiment 1 Trust Items. Participants rated three items on a five-point Likert scale (1 = Strongly Disagree, 5 = Strongly Agree), adapted from Lee and See [18]:

“The GenAI generated information is trustworthy.”

“The GenAI’s output is accurate.”

“The GenAI is well prepared to handle public health data.”

Experiment 2 Trust Items. The trust scale was expanded to six items by adding three items adapted from Gefen [19]:

“Even if not fully understood, I’d trust AI tools to do a good job,”

“I trust AI tools,” and

“AI tools are trustworthy.”

Following confirmatory factor analysis (CFA), items 1 (“The GenAI generated information is trustworthy”) and 4 (“Even if not fully understood, I’d trust AI tools to do a good job”) were removed due to weak standardized loadings (0.511 and 0.420, respectively; see Table D2c), yielding the final four-item scale (α = .796, CR = .800, AVE = .501) consisting of items 2, 3, 5, and 6: “The GenAI’s output is accurate,” “The GenAI is well prepared to handle public health data,” “I trust AI tools,” and “AI tools are trustworthy.”

**Experiment 1 GenAI Dependency Items**

Dependency was measured using the six items below (5-point Likert scale: Strongly disagree → Strongly agree), drawn from prior research on media and technology reliance [28]:

“I often use GenAI spontaneously.”

“I always prefer to use GenAI to find information.”

“My life is filled with conversations with GenAI.”

“GenAI is like an intimate friend.”

“I find it difficult to stop using GenAI.”

“I would feel uncomfortable without GenAI.”

**Experiment 2 GenAI Dependency Items**

The dependency scale was expanded to 17 items by retaining the six Experiment 1 items above and adding 11 items adapted verbatim from the Generative AI Dependency Scale validated by Goh et al. [17], rated on the same five-point Likert scale (1 = Strongly Disagree to 5 = Strongly Agree). The 11 items reflect the scale’s validated three-dimension structure:

**Cognitive Preoccupation (Salience and Compulsive Use)**

1. My decisions are often influenced by generative AI.
2. I feel an urge to use generative AI, even when it may not be necessary.
3. I look forward to using generative AI, even if it is unrelated to the task at hand.

**Negative Consequences**

4. My use of generative AI has caused concerns for me.
5. I have trouble completing work or other responsibilities without generative AI.
6. I feel less confident in my abilities without generative AI.
7. My use of generative AI has negatively affected my problem-solving skills or efficiency.

**Withdrawal**

8. I experience restlessness if I am unable to use generative AI.
9. I feel unsettled or distracted when I cannot use generative AI.
10. I feel a sense of disconnection when I cannot access generative AI.
11. I get frustrated or irritable when I am unable to use generative AI.

**APPENDIX D: Measurement Model**

**Experiment 1: Trust Scale (3 items)**

**Table S1. Fit Indices: Trust Scale (Experiment 1)**

| **Fit Index** | **Threshold** | **Value** |
| --- | --- | --- |
| χ² | -- | 0.000 |
| DoF | -- | 0 (just-identified) |
| CFI | ≥ 0.90 | 1.000 |
| RMSEA | ≤ 0.10 | N/A (just-identified) |

*Note. A three-item single-factor model is just-identified (df = 0); global fit indices are not interpretable. Results verified from Experiment 1 dataset (N = 338).*

**Table S2. Factor Loadings: Trust Scale (Experiment 1)**

| **Indicator** | **Std. Beta** | **SE** |
| --- | --- | --- |
| The GenAI generated information is trustworthy (T1) | 0.924 | N/A |
| The GenAI’s output is accurate (T2) | 0.935 | 0.054 |
| The GenAI is well prepared to handle public health data (T3) | 0.776 | 0.068 |

*Note. Standardized loadings computed from item correlations (r₁₂ = .863, r₁₃ = .717, r₂₃ = .726). SE not available for first indicator (fixed for identification). α = .909, CR = .912, AVE = .776. Results verified from Experiment 1 dataset (N = 338).*

**Experiment 1: Learned Dependency Scale (6 items)**

**Table S3. Fit Indices: Learned Dependency (Experiment 1)**

| **Fit Index** | **Threshold** | **Value** |
| --- | --- | --- |
| χ² | -- | 24.995 |
| DoF | -- | 9 |
| χ²/DoF | ≤ 5 | 2.777 |
| CFI | ≥ 0.90 | 0.977 |
| RMSEA | ≤ 0.10 | 0.068 |

*Note. Individual item-level dependency data not available in the current dataset; fit indices reported from original CFA analysis.*

**Table S4. Factor Loadings: Learned Dependency (Experiment 1)**

| **Indicator** | **Std. Beta** | **SE** |
| --- | --- | --- |
| GenAI preference_1 (I often use GenAI spontaneously) | 0.109 † | N/A |
| GenAI preference_2 (I always prefer to use GenAI to find information) | 0.489 † | 2.598 |
| GenAI preference_3 (My life is filled with conversations with GenAI) | 0.671 | 3.831 |
| GenAI preference_4 (GenAI is like an intimate friend) | 0.758 | 5.231 |
| GenAI preference_5 (I find it difficult to stop using GenAI) | 0.779 | 5.826 |
| GenAI preference_6 (I would feel uncomfortable without GenAI) | 0.782 | 5.433 |

*Note. † Loadings below 0.50. SE not available for first indicator. α = .782, CR = .786, AVE = .416. These values are reported from original CFA analysis.*

**Experiment 2: Trust Scale (4 items, CFA-reduced)**

**Table S5. Fit Indices: Trust Scale (Experiment 2)**

| **Fit Index** | **Threshold** | **Value** |
| --- | --- | --- |
| χ² | -- | 1.612 |
| DoF | -- | 2 |
| χ²/DoF | ≤ 5 | 0.806 |
| CFI | ≥ 0.90 | 1.000 |
| RMSEA | ≤ 0.10 | 0.000 |

**Table S6. Factor Loadings: Trust Scale (Experiment 2)**

| **Indicator** | **Std. Beta** | **SE** |
| --- | --- | --- |
| The GenAI’s output is accurate (Item 2) | 0.675 | N/A |
| The GenAI is well prepared to handle public health data (Item 3) | 0.748 | 0.071 |
| I trust AI tools (Item 5) | 0.701 | 0.061 |
| AI tools are trustworthy (Item 6) | 0.704 | 0.067 |

*Note. Items 1 and 4 removed due to standardized loadings of 0.511 and 0.420 respectively. SE not available for first indicator. α = .796, CR = .800, AVE = .501.*

**Experiment 2: Learned Dependency Scale (17 items)**

**Table S7. Fit Indices: Learned Dependency (Experiment 2)**

| **Fit Index** | **Threshold** | **Value** |
| --- | --- | --- |
| χ² | -- | 568.919 |
| DoF | -- | 119 |
| χ²/DoF | ≤ 5 | 4.781 |
| CFI | ≥ 0.90 | 0.915 |
| RMSEA | ≤ 0.10 | 0.082 |

**Table S8. Factor Loadings: Learned Dependency (Experiment 2)**

| **Indicator** | **Std. Beta** | **SE** |
| --- | --- | --- |
| GenAI preference_1 | 0.058 † | N/A |
| GenAI preference_2 | 0.370 † | 5.575 |
| GenAI preference_3 | 0.536 | 8.812 |
| GenAI preference_4 | 0.733 | 14.088 |
| GenAI preference_5 | 0.715 | 14.326 |
| GenAI preference_6 | 0.659 | 12.679 |
| GenAI preference_7 (Cognitive Preoccupation) | 0.507 | 7.952 |
| GenAI preference_8 (Cognitive Preoccupation) | 0.521 | 10.681 |
| GenAI preference_9 (Cognitive Preoccupation) | 0.618 | 10.882 |
| GenAI preference_10 (Negative Consequences) | 0.741 | 14.060 |
| GenAI preference_11 (Negative Consequences) | 0.780 | 15.441 |
| GenAI preference_12 (Negative Consequences) | 0.816 | 16.297 |
| GenAI preference_13 (Negative Consequences) | 0.781 | 16.551 |
| GenAI preference_14 (Withdrawal) | 0.813 | 17.284 |
| GenAI preference_15 (Withdrawal) | 0.787 | 16.915 |
| GenAI preference_16 (Withdrawal) | 0.818 | 18.472 |
| GenAI preference_17 (Withdrawal) | 0.804 | 16.432 |

*Note. † Loadings below 0.50. Items 1–6 = original Experiment 1 items. Items 7–17 = Goh et al. [17] scale; subscale labels in parentheses. SE not available for first indicator. α = .932, CR = .930, AVE = .462.*

**Reliability and Convergent Validity Summary**

**Table S9. Reliability and Convergent Validity Summary**

| **Construct** | **α** | **AVE** | **CR** | **√AVE** |
| --- | --- | --- | --- | --- |
| Trust (Exp 1, 3 items) | .909 | 0.776 | 0.912 | 0.881 |
| Learned Dependency (Exp 1, 6 items) | .782 | 0.416 | 0.786 | 0.645 |
| Trust (Exp 2, 4 items) | .796 | 0.501 | 0.800 | 0.708 |
| Learned Dependency (Exp 2, 17 items) | .932 | 0.462 | 0.930 | 0.680 |

*Note. AVE ≥ 0.50 and CR ≥ 0.70 are standard thresholds (Fornell and Larcker, 1981). Trust (Exp 1) values updated from CFA verified on Experiment 1 dataset (N = 338).*

**Discriminant Validity**

**Table S10. Discriminant Validity Assessment (Fornell-Larcker Criterion)**

| **Experiment** | **Inter-construct r (Trust–LD)** | **√AVE Trust** | **√AVE LD** | **Discriminant Validity** |
| --- | --- | --- | --- | --- |
| Experiment 1 (N=338) | 0.697 | 0.881 | 0.645 | Not supported (r > √AVE for LD construct) |
| Experiment 2 (N=563) | 0.643 | 0.708 | 0.680 | Supported (r < √AVE for both constructs) |

*Note. Discriminant validity holds when inter-construct r < √AVE for both constructs. In Experiment 1, r (0.697) exceeds √AVE for Learned Dependency (0.645), indicating the two constructs were not fully empirically separable with the shorter scales used in that experiment. This is acknowledged as a measurement limitation.*
