# Supplementary material for: Trust in Generative AI for Health Information Consumption and the Effect of Learned Dependency: Randomized Controlled Experimental Study
Source: J Med Internet Res. 2026 Sep 16;28:e98326. doi: 10.2196/98326 (PMC13580606; doi:10.2196/98326)
Supplement: Supplementary file 3 [file jmir-v28-e98326-s003.docx]

**CONSORT 2025 Checklist of Information to Include When Reporting a Randomized Trial**

Manuscript: Trust in Generative AI for Health Information Consumption and the Effect of Learned Dependency: Experimental Investigation

Manuscript #98326 | Journal of Medical Internet Research

*Note: This study is a behavioral online experiment, not a clinical trial. Several CONSORT items pertaining to clinical interventions, care providers, harms, and patient follow-up are not applicable (marked N/A with shading). Items are answered as fully as possible given the study design.*

| **Section / Topic** | **No** | **CONSORT 2025 checklist item description** | **Reported on page no.** |
| --- | --- | --- | --- |
| **Title and abstract** | | | |
| Title and structured abstract | **1a** | Identification as a randomised trial | Title (p. 1); Abstract Methods (p. 2) |
|  | **1b** | Structured summary of the trial design, methods, results, and conclusions | Abstract, pp. 1–2 |
| **Open science** | | | |
| Trial registration | **2** | Name of trial registry, identifying number (with URL) and date of registration | Not pre-registered. The study is a behavioral online experiment and was not entered into a clinical trial registry prior to data collection. This is acknowledged as a limitation. |
| Protocol and statistical analysis plan | **3** | Where the trial protocol and statistical analysis plan can be accessed | No pre-registered protocol. Analysis plan described in Data Analysis section (p. 13–14). Survey materials available as supplementary material. |
| Data sharing | **4** | Where and how the individual de-identified participant data (including data dictionary), statistical code and any other materials can be accessed | Data Availability Statement (p. 47). Data not publicly available per IRB Protocol STUDY00002235 (Exempt). Study and survey materials provided as supplementary material. |
| Funding and conflicts of interest | **5a** | Sources of funding and other support (e.g., supply of drugs), and role of funders in the design, conduct, analysis and reporting of the trial | Acknowledgements (p. 46). Funded by NIH/NLM R01LM011975. Funders had no role in design, conduct, analysis, or reporting. |
|  | **5b** | Financial and other conflicts of interest of the manuscript authors | Conflicts of Interest section (p. 47): None declared. |
| **Introduction** | | | |
| Background and rationale | **6** | Scientific background and rationale | Introduction, pp. 2–4; Theoretical Framework, pp. 4–6 |
| Objectives | **7** | Specific objectives related to benefits and harms | Introduction (p. 4), Research Questions RQ1–RQ2; Hypotheses H1–H5 (pp. 6–8) |
| **Methods** | | | |
| Patient and public involvement | **8** | Details of patient or public involvement in the design, conduct and reporting of the trial | N/A |
| Trial design | **9** | Description of trial design including type of trial (e.g., parallel group, crossover), allocation ratio, and framework (e.g., superiority, equivalence, non-inferiority, exploratory) | Methodology (p. 9); Experiment Design (pp. 9–10). Two parallel-group 2×2 between-subjects factorial RCTs (superiority framework). Participants randomly assigned 1:1:1:1 across 4 conditions × 5 health texts = 20 groups. |
| Changes to trial protocol | **10** | Important changes to the trial after it commenced including any outcomes or analyses that were not prespecified, with reason | No pre-registered protocol existed. The three-way interaction term (Accuracy × TVC × Learned Dependency, H5) and the expansion of the dependency scale from 6 to 17 items in Experiment 2 were design modifications implemented before data collection for Experiment 2. The exploratory three-way interaction analysis was added based on theoretical reasoning (pp. 7–8). |
| Trial setting | **11** | Settings (e.g., community, hospital) and locations (e.g., countries, sites) where the trial was conducted | Experiment 1: University of Arizona, online via Qualtrics, business course (p. 9). Experiment 2: Amazon Mechanical Turk, US-based workers, online via Qualtrics (p. 12). |
| Eligibility criteria | **12a** | Eligibility criteria for participants | Experiment 1: Junior-level business students enrolled in a university course (p. 9). Experiment 2: US-based MTurk workers with ≥95% approval rating (p. 12). |
|  | **12b** | If applicable, eligibility criteria for sites and for individuals delivering the interventions (e.g., surgeons, physiotherapists) | N/A |
| Intervention and comparator | **13** | Intervention and comparator with sufficient details to allow replication. If relevant, where additional materials describing the intervention and comparator (e.g., intervention manual) can be accessed | Stimuli section (pp. 10–11, 13). Interventions: (1) Information accuracy: correct vs. incorrect GPT-4o-generated health text (3 altered locations in Exp1; 6 in Exp2); (2) TVC: bold-text highlighting vs. no highlighting. Source texts: MIMIC-III discharge summaries. Supplementary materials include full item lists. |
| Outcomes | **14** | Pre-specified primary and secondary outcomes, including the specific measurement variable (e.g., systolic blood pressure), analysis metric (e.g., change from baseline, final value, time to event), method of aggregation (e.g., median, proportion), and time point for each outcome | Measures sections (pp. 11, 13–14). Primary outcome: Trust in AI-generated health information (3-item Likert scale in Exp1; 4-item in Exp2; 1–5 scale; mean composite score). Measured at single post-exposure time point. No secondary outcomes. |
| Harms | **15** | How harms were defined and assessed (e.g., systematically, non-systematically) | N/A |
| Sample size | **16a** | How sample size was determined, including all assumptions supporting the sample size calculation | No formal a priori power analysis was conducted. Experiment 1 used a convenience sample of enrolled students (N=338). Experiment 2 used MTurk recruitment (N=563). Sample sizes are reported in Demographics section (p. 14). See Limitations (p. 44). |
|  | **16b** | Explanation of any interim analyses and stopping guidelines | N/A |
| **Randomization** | | | |
| Sequence generation | **17a** | Who generated the random allocation sequence and the method used | Experiment Design (pp. 9–10, 12–13). Random allocation sequence generated automatically by Qualtrics using built-in randomization engine. |
|  | **17b** | Type of randomisation and details of any restriction (e.g., stratification, blocking and block size) | Simple randomization via Qualtrics; no blocking or stratification. Participants assigned to one of 20 experimental groups (2 accuracy × 2 TVC × 5 health texts). |
| Allocation concealment mechanism | **18** | Mechanism used to implement the random allocation sequence (e.g., central computer/telephone; sequentially numbered, opaque, sealed containers), describing any steps to conceal the sequence until interventions were assigned | Qualtrics automated randomization at point of survey entry. Participants were unaware of condition assignment. Back-navigation disabled to prevent re-assignment. |
| Implementation | **19** | Whether the personnel who enrolled and those who assigned participants to the interventions had access to the random allocation sequence | Allocation performed automatically by Qualtrics. Research team did not manually assign participants. No personnel had access to or could influence the allocation sequence. |
| Blinding | **20a** | Who was blinded after assignment to interventions (e.g., participants, care providers, outcome assessors, data analysts) | Participants were not informed of their condition assignment (implicit blinding). No care providers involved. Outcome assessors: trust measured via self-report immediately after stimulus exposure; no separate outcome assessor. |
|  | **20b** | If blinded, how blinding was achieved and description of the similarity of interventions | N/A |
| Statistical methods | **21a** | Statistical methods used to compare groups for primary and secondary outcomes, including harms | Data Analysis (pp. 13–14). Hierarchical multiple regression (HMR) with three blocks. Main effects and interaction effects tested via OLS regression in R 4.2.1. |
|  | **21b** | Definition of who is included in each analysis (e.g., all randomised participants), and in which group | Data Analysis (p. 13–14). All participants who completed the survey with no missing data on study variables included (listwise deletion). Final N=338 (Exp1), N=563 (Exp2). Analysed in the group to which they were randomly assigned. |
|  | **21c** | How missing data were handled in the analysis | Listwise deletion. Qualtrics forced response minimized item-level missingness. Data Availability Statement (p. 47). |
|  | **21d** | Methods for any additional analyses (e.g., subgroup and sensitivity analyses), distinguishing prespecified from post-hoc | Data Analysis (p. 13–14). CFA conducted to assess psychometric properties (prespecified). Exploratory three-way interaction (H5: Accuracy × TVC × LD) was theoretically motivated prior to data collection. Subscale analysis (Appendix E) is post-hoc exploratory. |
| **Results** | | | |
| Participant flow, including flow diagram | **22a** | For each group, the numbers of participants who were randomly assigned, received intended intervention, and were analysed for the primary outcome | See CONSORT Flow Diagram (separate document). Demographics (p. 14). Exp1: N=338 assigned and analysed. Exp2: N=563 assigned and analysed. |
|  | **22b** | For each group, losses and exclusions after randomisation, together with reasons | See CONSORT Flow Diagram. Participants with missing data excluded via listwise deletion prior to analysis. Exact numbers of excluded participants not available from the current dataset; this is acknowledged as a limitation. |
| Recruitment | **23a** | Dates defining the periods of recruitment and follow-up for outcomes of benefits and harms | Both experiments conducted in 2024. Specific dates available from the corresponding author upon request. No follow-up (single time-point design). |
|  | **23b** | If relevant, why the trial ended or was stopped | N/A |
| Intervention and comparator delivery | **24a** | Intervention and comparator as they were actually administered (e.g., where appropriate, who delivered the intervention/comparator, how participants adhered, whether they were delivered as intended [fidelity]) | Procedure sections (pp. 11, 13). Delivered via Qualtrics online survey. All participants completed the intervention as intended (online self-administered). No deviation from protocol. |
|  | **24b** | Concomitant care received during the trial for each group | N/A |
| Baseline data | **25** | A table showing baseline demographic and clinical characteristics for each group | Table 1: Demographic information (p. 15). Includes gender, age, race, ethnicity, education, and major field of study for both experiments. |
| Numbers analysed, outcomes and estimation | **26** | For each primary and secondary outcome, by group:   - the number of participants included in the analysis - the number of participants with available data at the outcome time point - result for each group, and the estimated effect size and its precision (such as 95% confidence interval)   for binary outcomes, presentation of both absolute and relative effect size | Results — Regression section (pp. 16–19). Table 2: Regression Results (p. 17). Table 3: Hypothesis Testing Results (p. 20). Effect sizes (B) and 95% CIs reported for all conditions. |
| Harms | **27** | All harms or unintended events in each group | N/A |
| Ancillary analyses | **28** | Any other analyses performed, including subgroup and sensitivity analyses, distinguishing pre-specified from post-hoc | Reliability & Validity (pp. 14–15). CFA reported in Multimedia Appendix D. Exploratory subscale analysis reported in Multimedia Appendix E (post-hoc). |
| **Discussion** | | | |
| Interpretation | **29** | Interpretation consistent with results, balancing benefits and harms, and considering other relevant evidence | Discussion — Principal Findings (pp. 39–42). |
| Limitations | **30** | Trial limitations, addressing sources of potential bias, imprecision, generalisability, and, if relevant, multiplicity of analyses | Limitations section (pp. 43–45). Nine limitations reported including sample representativeness, manipulation check absence, ecological validity, self-report bias, discriminant validity concern in Exp1, and scope of TVC manipulation. |

**We strongly recommend reading this statement in conjunction with the CONSORT 2025 Explanation and Elaboration and/or the CONSORT 2025 Expanded Checklist for important clarifications on all the items. We also recommend reading relevant CONSORT extensions. See www.consort-spirit.org.*

*Citation: Hopewell S, Chan AW, Collins GS, Hróbjartsson A, Moher D, Schulz KF, et al. CONSORT 2025 Statement: updated guideline for reporting randomised trials. BMJ. 2025; 388:e081123. https://dx.doi.org/10.1136/bmj-2024-081123.*

*© 2025 Hopewell et al. This is an Open Access article distributed under the terms of the Creative Commons Attribution License (https://creativecommons.org/licenses/by/4.0/), which permits unrestricted use, distribution, and reproduction in any medium, provided the original work is properly cited.*
